# Introducing a Porcine Inflammatory Ex Vivo Retina Model for Diabetic Retinopathy

**DOI:** 10.3390/ijms26083919

**Published:** 2025-04-21

**Authors:** Agnes Mühle, Sven Schnichels, José Hurst

**Affiliations:** Section for Translational Research in Ophthalmology, Centre for Ophthalmology, University Eye Hospital Tübingen, Elfriede-Aulhorn-Str. 7, 72076 Tübingen, Germany; agnes.muehle@med.uni-tuebingen.de (A.M.); jose.hurst@med.uni-tuebingen.de (J.H.)

**Keywords:** inflammation, diabetic retinopathy, ex vivo model, porcine, TNF-a, IL-6, neoangiogenesis

## Abstract

This study aimed to develop an ex vivo retinal model to examine inflammatory processes in diabetic retinopathy (DR) without animal testing. Porcine eyes were collected from a local abattoir, dissected, and cultivated for four days in five experimental groups: control group (Co), 25 mM and 50 mM mannitol groups (Man25, Man50) as osmotic controls, and 25 mM and 50 mM glucose groups (Glc25, Glc50) as diabetic groups. A TUNEL assay was used to determine relative cell death. Immunofluorescence and quantitative real-time polymerase chain reaction (qRT-PCR) were performed to detect inflammatory markers. An increase in the cell death rate in Man50 (30%), Glc25 (36%) and Glc50 (37%) compared to Co (12%) (*p* < 0.01, *p* < 0.001, *p* < 0.001, respectively) and between Glc25 and Man25 (21%) (*p* < 0.01) was found. Immunofluorescence staining and qRT-PCR analysis revealed a TNF-α increase in Glc25 compared to Man25 and Co. iNOS was increased in Glc25 vs. Man25 but not in Co vs. Glc25. *iNOS* gene expression was upregulated with Glc25 treatment compared to Co and Man25 groups. Expression levels of *IL-6* and *CD31* were significantly higher in Glc25 than in Co and Man25. Glucose treatment increased cell death and inflammation, prompting us to present a DR model for better understanding DR and testing new therapies.

## 1. Introduction

Diabetic retinopathy (DR) is a common long-term complication of diabetes mellitus (DM) and one of the leading causes of vision loss in midlife [1].

### 1.1. Pathogenesis

**Figure 1:**
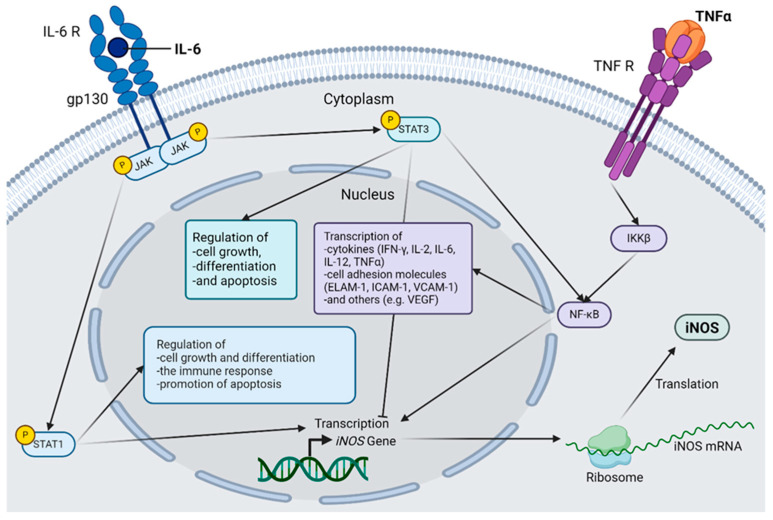
Selected inflammatory cascades in diabetic retinopathy. The connection between interleukin(IL)-6, tumor necrosis factor α (TNF-α) and inducible nictric oxide synthetase (iNOS) is shown. IL-6 activates Janus kinases (JAK), which in turn activate both signal transducer and activator of transcription (STAT) 1 and STAT3 via the IL-6 receptor, forming a hexamer together with glycoprotein 130 (gp130). STAT1 acts as a transcription factor for iNOS and regulates cell growth and differentiation, as well as the immune response. STAT1 also has a proapoptotic effect. STAT3 also regulates cell growth, differentiation, and apoptosis but can have both pro- and anti-apoptotic effects. STAT3 directly activates the nuclear factor kappa-light-chain-enhancer of activated B cells (NF-κB) and simultaneously inhibits iNOS transcription. TNF-α binds to its corresponding receptor, and the inhibitor of nuclear factor kappa-B kinase subunit beta (IKKβ) is activated via several intermediate steps that are necessary for the activation of the transcription factor NF-κB. The genes induced by NF-κB are mainly inflammatory mediators, including iNOS; cytokines, such as Interferon-gamma (IFN-γ), IL-2, IL-6, IL-12, and TNF-α; various cell adhesion molecules, such as endothelial-leukocyte adhesion molecule 1 (ELAM-1), Intercellular Adhesion Molecule 1 (ICAM-1), and vascular cell adhesion protein 1 (VCAM-1); and other factors, such as vascular endothelial growth factor (VEGF). After the induction of *iNOS* gene transcription, the mRNA is translated to the ribosome, and the protein is produced, which plays an important role in the pathogenesis of diabetic retinopathy. Abbreviations: gp130, glycoprotein 130; IKKβ, inhibitor of the enhancer of the kappa light polypeptide gene in B cells beta; IL, interleukin; iNOS, inducible nitric oxide synthetase; JAK, Janus kinase; mRNA, messenger RNA; NF-κB, nuclear factor kappa light chain enhancer of activated B cells; R, receptor; STAT, Signal Transducers and Activators of Transcription; TNF, tumor necrosis factor. The figure was created by using https://www.biorender.com/ accessed 12 December 2024.

The disruption of microvascularization is a well-understood area of research in DR [2]. Current DR research indicates that immunological processes are significantly involved in disease development, although the exact pathogenesis remains unclear [3]. Due to altered metabolism and vascular dysfunction, Müller cells and microglia produce glutamate, inducible nitric oxide synthase (iNOS), and pro-inflammatory cytokines, such as interleukin-1β (IL-1β), interleukin-6 (IL-6), interleukin-12 (IL-12), and tumor necrosis factor α (TNF-α), which lead to exacerbated cell death of retinal cells by the activation of caspases (Figure 1) [4,5,6]. TNF-α and IL-6 act through the nuclear factor kappa light chain enhancer of the activated B cell (NF-κB) pathway as mediators of iNOS expression [7]. These proinflammatory cytokines regulate apoptosis, cell differentiation and cell growth as well as neoangiogenesis. However, the inflammatory hypothesis is currently insufficient to explain all pathological changes in DR, especially because other inflammatory conditions do not cause diabetes-like retinopathy in non-diabetic patients or animals [8]. For instance, retinal leukostasis has been observed in insulin-resistant animals without diabetes that do not develop diabetes-like retinopathy. Expansion of our understanding of DR and its pathogenesis is, therefore, needed, and neuronal cell death, inflammatory processes, and impairment of the neurovascular unit should be considered when developing new therapeutic strategies for DR [3].

### 1.2. Ex Vivo Models

Despite the continued need for patient research, models that allow hypothesis-driven research are needed [9]. In vivo models should be reduced because of animal welfare concerns [10,11]. A disadvantage of organ cultures is the lack of tissue embedding in the organism, which is characterized by a lack of blood flow that limits the cultivation time [9,12]. Retinal explants are best suited to resemble in vivo conditions, with preservation of the complex neuronal and non-neuronal cellular connections of the retina [13,14]. To date, there is no established DR model that correctly represents all aspects and processes of the human eye [9,15].

Only a few ex vivo retinal models for DR exist, such as those introduced by Knott et al. 1999 or Louie et al. 2021 using the human retina [16,17]. Furthermore, there are ex vivo models of retinas obtained from rodents and subsequently treated with glucose [18,19,20].

### 1.3. Different Species

The goal is to obtain ex vivo material from animals that reflect human retinal morphology and physiology as accurately as possible [13]. Pigs are used for research purposes in various medical disciplines because of their anatomical and morphological similarity [10]. Human and porcine eyes also show clear homologies, especially in terms of size, retinal structure, and anatomy, making them suitable organisms for a retinal DR model [21,22,23]. A key anatomical difference is vascularization; while human eyes are supplied by one central artery, pigs have four retinal arterial branches originating from the ciliary artery [10,24]. Characteristic lesions of DR, such as exudates, hemorrhages, and cotton wool spots, also occur rarely in diabetic rodent models but do occur in pig, dog, and monkey models [15].

The advantages of using pig eyes outweigh the disadvantages owing to their anatomical and morphological similarities to the human retina as well as the homologous formation of diabetic retinal lesions.

### 1.4. Research Question

Other research groups have shown elevated cell death and neovascularization in retinal explants from mice, rats, and humans treated with glucose [16,17,18,19,20].

The hypothesis of this study is that treatment of porcine retinal explants with glucose damages different cell types in the retina and provokes a measurable immune response. The aim of this study was to find a suitable ex vivo retinal model that represents the inflammatory processes in the retina during DR without the need for killing animals.

## 2. Results

### 2.1. Glucose Treatment-Induced Cell Death

**Figure 2:**
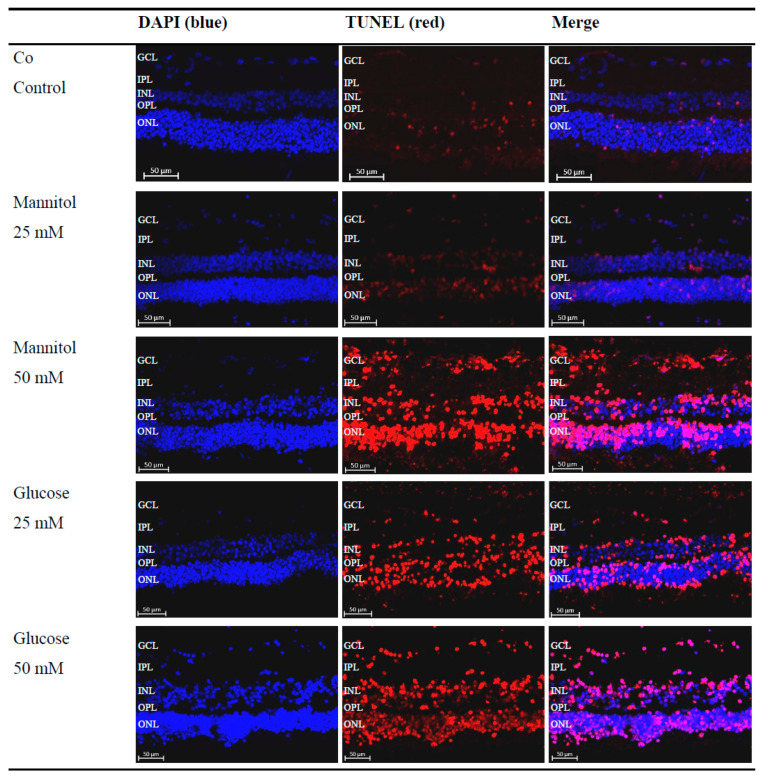
Increased detection of terminal deoxynucleotidyl transferase dUTP nick-end labeling (TUNEL)+ cells after glucose treatment. Representative microscopic images of porcine retinal explants with nuclear staining (4′-6-diaminodino-2-phenylindole (DAPI), blue), staining of apoptotic cells (TUNEL, red), which were treated with the indicated stressors for four days. An increased red signal compared to that of the control (Co) and 25 mM D-Mannitol group (Man25) in the 50 mM D-Mannitol group (Man50), 25 mM D-Glucose group (Glc25), and 50 mM D-Glucose group (Glc50) groups was evident. Abbreviations: DAPI, 4′-6-diaminodino-2-phenylindole; GCL, ganglion cell layer; INL, inner nuclear layer; IPL, inner plexiform layer; ONL, outer nuclear layer; OPL, outer plexiform layer; TUNEL, terminal deoxynucleotidyl transferase dUTP nick-end labeling.

By treating the retinal explants with glucose solution, a hyperglycemic metabolic state of the retina is simulated, as occurs in DR. To evaluate the effects of high-glucose treatment on retinal explants, apoptosis was analyzed using the terminal deoxynucleotsidyl transferase dUTP nick-end labeling (TUNEL) assay. After performing the TUNEL assay, all 4′-6-diaminodino-2-phenylindole(DAPI) + -labeled nuclei and TUNEL+ cells were counted, and the ratio of TUNEL+ cells to the total cell number was calculated and divided by cell layer (Figure 2).

**Figure 3:**
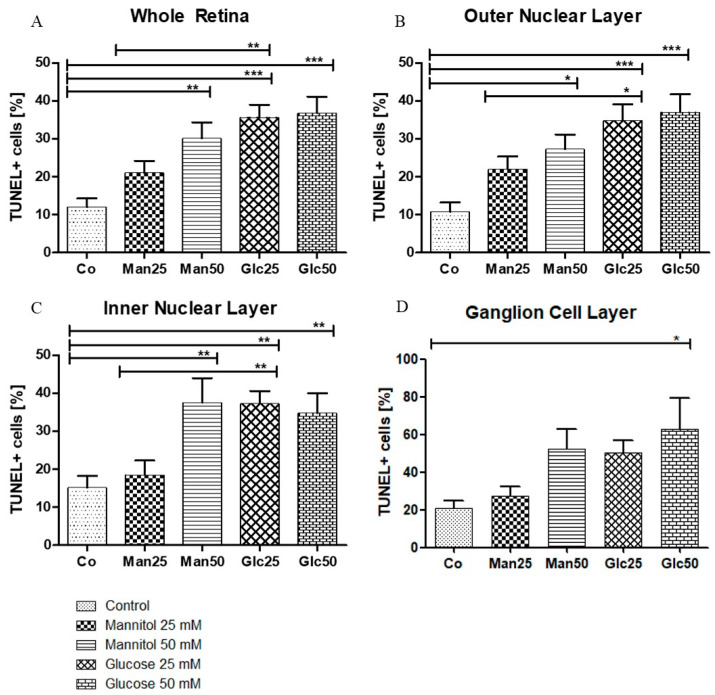
Glucose treatment induces apoptosis. The percentage of TUNEL+ cells (**A**) is displayed in relation to the entire retina and individual cell layers ((**B**) outer nuclear layer; (**C**) inner nuclear layer; (**D**) ganglion cell layer). The tissue was treated with the indicated stressors for four days. In (**A**–**C**), there were significantly more apoptotic cells in the Man25, Glc25, and Glc50 groups than in the control group. Statistical differences were also found in (**A**–**C**) between the Man25 and Glc25 groups but not between Man50 and Glc50. A significantly higher proportion of apoptotic cells was detected in the Glc50 group (63%) than in the control group (21%). No other statistical differences were found in the ganglion cell layer (**D**). Shown is the mean ± SEM, *n* ≥ 26. Significance levels * *p* < 0.05, ** *p* < 0.01, *** *p* < 0.001. Abbreviations: Co, control; Man, mannitol; Glc, glucose; TUNEL, terminal deoxynucleotidyl transferase dUTP nick-end labeling.

A significant increase in apoptosis was observed between the control group (Co) (12%) and 25 mM D-Glucose group (Glc25) group (36%) (*p* < 0.001) and between the 25 mM D-Mannitol group (Man25) (21%) and Glc25 groups (*p* < 0.01) for the whole retina. A comparison of the control with the 50 mM D-Mannitol group (Man50) (30%) and the 50 mM D-Glucose group (Glc50) (37%) showed a significant increase in cell death (both *p* < 0.01 and *p* < 0.001), but no significant difference was observed between Man50 and Glc50 (Figure 3A). In the outer nuclear layer (ONL), 11% of TUNEL+ cells in the control group were statistically significant compared to 35% of TUNEL+ cells in the Glc25 group (*p* < 0.001). There was a significantly lower apoptosis rate in the Man25 group (22% TUNEL+ cells) compared to that in the Glc25 group (*p* < 0.05). The Man50 (27%) and Glc50 (37%) groups had more apoptotic cells than the control group (*p* < 0.05, *p* < 0.001), but there was no significant difference between them (Figure 3B). Similarly, in the inner nuclear layer (INL), Glc25 (37%) showed significantly more labeled cells than the control (15%) (*p* < 0.01) and Man25 (19%) (*p* < 0.01), and Man50 (38%) and Glc50 (35%) were different from the control (both *p* < 0.01) (Figure 3C). Within the individual cell layers, the highest percentage of cell death was recorded in the ganglion cell layer (GCL): the control group had the lowest value (21%), followed by Man25 (27%), Glc25 (50%), Man50 (52%), and Glc50 (63%), with only a significant difference between Glc50 and the control group (*p* < 0.05) (Figure 3D).

Except for the GCL, all cell layers and the whole retina were significantly different between the native and osmotic control groups and the Glc25 group, whereas no differences were observed between Glc50 and its corresponding osmotic control group. As no change in apoptosis was recorded between the Man50 and Glc50 groups, an osmotic effect was likely the predominant reason for cell impairment. Therefore, these groups were excluded from further investigations because of the limited significance to the research question.

In summary, the lowest rates of apoptosis were detected in the control group, and the highest rates were observed for Glc25, Man50, and Glc50. Broken down by cell layers, the largest proportion of TUNEL+ cells was detected in the GCL with a maximum of 63%, while the lowest proportion was found in the ONL (11%).

### 2.2. Treatment with Glucose-Induced Retinal Inflammation

After confirming the increased number of apoptotic cells, changes in inflammatory markers were evaluated via histology and quantitative real-time PCR (qRT-PCR). In immunofluorescence (IF), it is not only possible to quantify the signal but also to determine the exact localization of markers. qRT-PCR, on the other hand, is well suited for quick and precise testing of the relative quantity of different markers. We wanted to confirm that inflammatory markers are increasingly expressed as a result of hyperglycemia.

**Figure 4:**
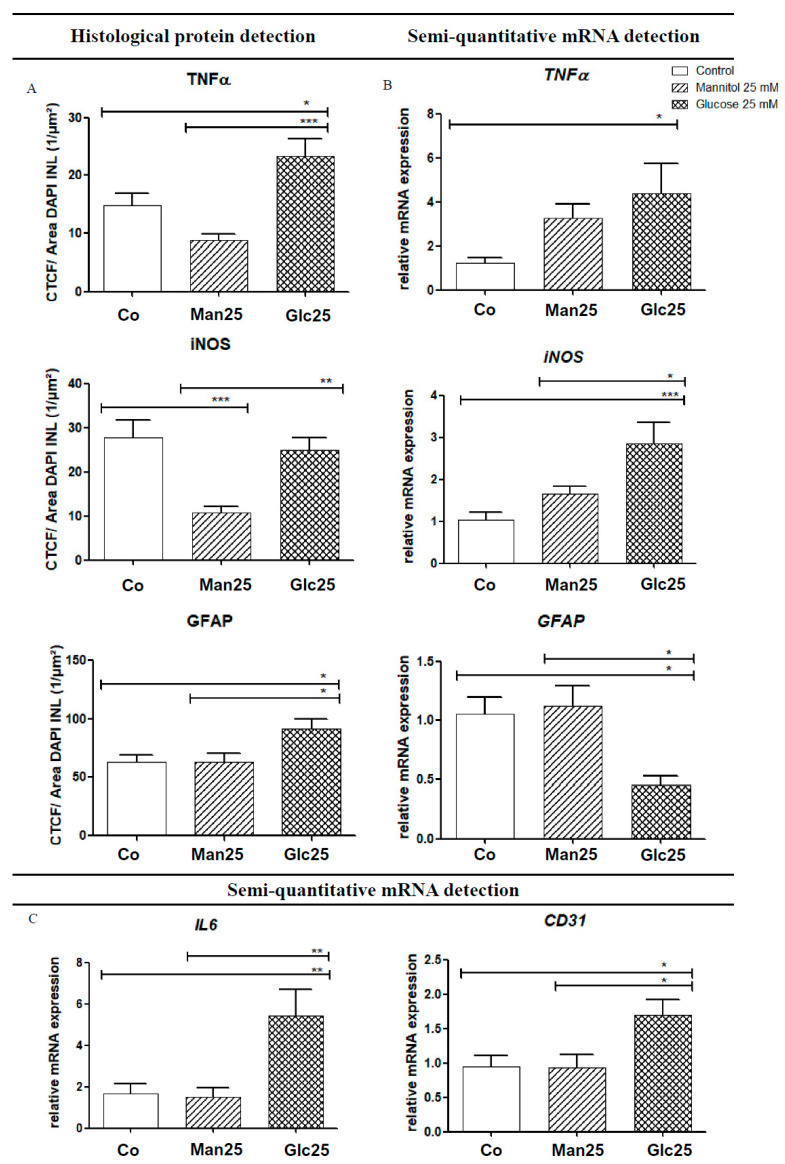
Increased protein levels (**A**) and gene expression (**B**,**C**) of various inflammatory markers following treatment with glucose. Translation (**A**) and expression (**B**,**C**) of various inflammatory markers, as well as the glucose group, are shown for native and osmotic controls. Immunofluorescence (IF) detection (**A**) corresponds to mRNA detection using quantitative real-time polymerase chain reaction (qRT-PCR) (**B**). For IL-6 and cluster of differentiation 31 (CD31), only gene transcription was analyzed (**C**). The tissue was treated with the indicated stressors for four days. In TNF-α IF, an increase in the glucose group was detected compared to the controls, whereas a 3.6-fold increase in *TNF-α* mRNA was observed in Glc25 compared to the control. A significant reduction in iNOS was observed in the Man25 group compared to the control group by IF, and a significant increase in Glc25 compared to Man25, with no difference between the control and glucose groups. *iNOS* mRNA analysis showed a significant increase in the glucose group compared with that in the control group. Histologically, there was a significant increase in glial fibrillary acidic protein (GFAP) expression in the glucose group compared with that in both control groups. Using qRT-PCR, reduced expression of *GFAP* mRNA was detected in Glc25 compared to that in both controls. The expression of *IL-6* was increased by 3.2-fold (compared to the control) or 3.6-fold (compared to Man25) in Glc25. *CD31* mRNA expression increased in the glucose group compared to that in the Man25 group (1.8-fold). Shown is the mean ± SEM, *n* ≥ 10. Significance levels * *p* < 0.05, ** *p* < 0.01, *** *p* < 0.001. Abbreviations: CD31, Cluster of differentiation 31; Co, control; Man, mannitol; GFAP, glial fibrillary acidic protein; Glc, glucose; IL, Interleukin; iNOS, inducible nitric oxide synthetase; TNF, tumor necrosis factor.

As an activator of the NF-κB pathway, TNF-α acts as a mediator for the transcription of various cytokines (IL-6, iNOS, etc.) and adhesion molecules (vascular endothelial growth factor (VEGF), intercellular adhesion molecule 1 (ICAM-1), etc.). Histologically, TNF-α levels were significantly higher in the glucose group than in the osmotic control group (*p* < 0.001) and the control group (*p* < 0.05). In contrast, qRT-PCR analysis of *TNF-α* mRNA revealed a statistically significant upregulation (by 3.6-fold) of mRNA in the Glc25 group compared to that in the control group (*p* < 0.05). There were no statistically significant differences between the osmotic control and glucose-treated groups (Figure 4A,B).

IF analysis of iNOS, a factor that influences neoangiogenesis, vascular tone and insulin secretion, and whose transcription is mediated by IL-6 and TNF-α, showed a statistically significant increase between the Man25 group and the corresponding glucose group (*p* < 0.005), but not between the glucose treatment group and the control group. In addition, there was a significant decrease from the control to the osmotic control (*p* < 0.001). The mRNA expression of *iNOS* increased 2.8-fold in the glucose treatment compared to that in the control (*p* < 0.001) and 1.7-fold in the osmotic control (*p* < 0.05). A significant increase in *iNOS* mRNA compared to that in the Man25 group was confirmed by IF. A decrease in iNOS in the osmotic control group compared to that in the control group, as observed by IF, was not recovered by qRT-PCR (Figure 4A,B).

*IL-6* interacts with various downstream metabolic pathways that regulate apoptosis, cell differentiation and the immune response and it also influences the transcription of *iNOS.* Its mRNA expression was significantly increased by 3.2-fold in the Glc25 group compared to that in the control group and 3.6-fold compared to that in the osmotic control group (both *p* < 0.01) (Figure 4C).

### 2.3. Effect of Glucose Treatment on Endothelial Cells and Müller Cells

The expression of the endothelial cell marker *cluster of differentiation 31* (*CD31*) was significantly increased in the Glc25 group compared to that in both control groups (1.8-fold, *p* < 0.05) (Figure 4C).

Regarding glial fibrillary acidic protein (GFAP), a Müller cell marker, in IF, a significant increase was observed in the glucose-handling group compared to that in the control (*p* < 0.05) and osmotic control (*p* < 0.05) groups. However, via qRT-PCR, *GFAP* mRNA expression decreased (*p* < 0.05) in Glc25 compared to that in the control (2.3-fold) and Man25 (2.5-fold) (Figure 4A,B).

In summary, the ex vivo model demonstrated an increased apoptosis rate under glucose treatment across the entire retina. All tested inflammatory markers were more detectable in the glucose-treated experimental group and the endothelial cell marker CD31 as an indicator of neoangiogenesis, which was also increasingly expressed during treatment. The osmotic control meant that purely osmotic effects could be virtually ruled out.

## 3. Discussion

TUNEL assay demonstrated widespread cell damage to the retina, particularly in the inner and outer nuclear layers (Figure 3). It is essential to differentiate between osmotic and diabetic impairment in increased apoptosis; however, no increase in apoptosis was observed in the osmotic control group treated with the 25 mM solution. In contrast, apoptosis was observed in glucose-treated retinal explants, indicating pathological changes resulting from the unique properties of glucose. In the 50 mM group, there was no significant difference between the diabetic and osmotic control groups, suggesting that osmotic damage was predominant. Therefore, these groups were excluded from further analyses. The results show that cell damage could be caused by hyperglycemia in the ex vivo model.

Similar results were shown by Valdes et al. 2016 in murine retinal lances: The Glc25 group largely corresponds to the high-glucose, no-insulin group in which an increased occurrence of TUNEL-positive cells in the INL and ONL was also observed [19]. The retinal explants were treated with 34 mM glucose for four days in Valdes’ case [19], corresponding to the treatment carried out here with 25 mM glucose solution for four days. Therefore, it can be concluded that treatment of retinal explants with glucose causes cell damage across species, resulting in programmed cell death, particularly in the inner and outer nuclear layers.

Interestingly, in the publication by Valdes et al., there was no separate consideration of the GCL [19], whereas other publications have shown that ganglion cells are the first cells to be damaged in a diabetic metabolic state [25]. In the experiments performed in this study, no increase in apoptosis was detected in the GCL when treated with glucose compared with the controls. Nevertheless, the GCL had the largest proportion of TUNEL+ cells. In the control group, an average of 21% apoptotic nuclei were detected, which was 1.75 times more than when considering the Co of the whole retina (12%) (Figure 3). The cells were likely already damaged, and glucose appeared to have little effect on cell death. This finding aligns with that of Schnichels et al. (2017) [26].

Additionally, Oshitari et al. [18] highlighted the importance of these findings by comparing retinal ganglion cell damage in diabetic rats with ex vivo retinal lances treated with glucose for seven days. The cells were probably pre-damaged, and glucose contributed little to further cell death. Consistently, damage to ganglion cells by explantation was observed in a 2017 publication by Schnichels et al. [26].

The significance of the results is underlined by a study conducted by Oshitari and colleagues in 2014: This compared the retina of diabetic rats with ex vivo retinal lances treated with glucose for 7 days [18]. TUNEL analysis showed a similar number of apoptotic cells in the GCL in the ex vivo glucose group and in the DM group compared to the normal and osmotic control groups [18], suggesting similar results between ex vivo treatment with glucose and in vivo manifest diabetes. The study proved that the number of apoptotic cells was comparable in the glucose group and in the retinal explant group of diabetic rats, but that neurites regenerated better in the low-glucose group than in the high-glucose ex vivo group or in theDM group [18]. The findings of this study suggest a substantial overlap between ex vivo and in vivo experiments conducted on animal models. This suggests a strong association between the data obtained from ex vivo and in vivo models.

The results of this study are consistent with the scientific context and suggest cross-species apoptosis in the treatment of retinal lances using glucose or in diabetic test animal retinas.

### 3.1. Inflammation

When interpreting the results of qRT-PCR and immunofluorescence analyses, it is crucial to consider the distinct time points at which the observations were made. While qRT-PCR provides a quantitative measurement of the mRNA present in the tissue, IF assesses protein abundance. Consequently, the findings from these two methods may contradict each other, which can be taken as an indication of a modified time course.

Upon glucose treatment, TNF-α was elevated in IF compared to the osmotic and native controls. However, qRT-PCR analysis showed that *TNF-α* mRNA levels were higher in the Glc25 group than in the control group, with no discernible differences between the Glc25 and osmotic control groups. These results suggested that an increased concentration of TNF-α was consistently present in the glucose group in the ex vivo model. The lack of difference from osmotic control in qRT-PCR suggests that osmotic processes are involved in this upregulation. However, this cannot be considered the sole cause because no difference was observed between the control and osmotic control groups. These results do not allow for a conclusive interpretation of TNF-α expression. In summary, overexpression of TNF-α was observed in the Glc25 group, which was consistent with the findings of increased cytokine secretion in DR. The extent of the osmotic component in TNF-α overexpression needs to be clarified in further experiments. There are indications that osmosis in DR also influences homeostasis; however, further research is required to make conclusive conclusions [27]. Combining the results of IF and qRT-PCR, it seems plausible that the glucose concentration triggered the overexpression of TNF-α. Over time, osmotic changes may contribute to the upregulation of TNF-α.

However, since no difference was observed between the native and osmotic control groups, osmosis alone does not fully explain the increased TNF-α expression. This suggests a synergistic effect, where glucose-induced hyperosmolarity may act together with metabolic changes unique to glucose metabolism to enhance TNF-α expression. qRT-PCR measures mRNA levels and thus reflects transcriptional activity, IF provides protein abundance, which is also influenced by post-transcriptional and post-translational regulation, including mRNA stability, translational efficiency, and protein degradation rates. It is possible that, although osmotic stress in retinal tissue may not significantly increase TNF-α mRNA expression beyond glucose treatment levels, it could still affect protein synthesis or stabilization, thereby influencing the IF signal. Further studies could investigate the impact of a hyperosmolar metabolic state on TNF-α expression.

Using IF staining, significantly more iNOS was detected in the control and glucose groups than in the osmotic control group. The results are not unambiguous; the protective effect of mannitol could be discussed, but this cannot be adequately explained pathophysiologically. In contrast to the IF results, qRT-PCR showed a clear overexpression of *iNOS* RNA in Glc25 compared to the control and osmotic control.

Based on the present study, upregulation of *iNOS* mRNA fits into the existing picture of an inflammatory reaction in DR [28].

In our experiments, iNOS expression was elevated in both qRT-PCR and immunofluorescence (IF) assays in the Glc25 group compared to the osmotic control group, supporting the notion that glucose-specific metabolic pathways contribute to the overproduction of iNOS, beyond mere osmotic effects.

This observation suggests that high glucose levels may activate inflammatory signaling cascades, such as the NF-κB pathway, which are known to transcriptionally regulate iNOS expression. The increase in both mRNA and protein levels in the Glc25 group strengthens the hypothesis that hyperglycemia directly induces iNOS upregulation in the ex vivo model.

Interestingly, histological analysis revealed a reduced IF signal for iNOS in the mannitol (osmotic control) group compared to the native control, which may point to a more nuanced osmotic effect—potentially involving suppression of iNOS translation or altered protein stability under osmotic stress. Furthermore, the lack of a significant difference between the glucose and native control groups in IF could indicate regional variability in iNOS expression within the tissue or limitations in IF sensitivity and quantification.

To resolve these ambiguities and better quantify iNOS protein levels, Western blotting should be employed in future studies. This technique would allow for more accurate protein-level comparisons across groups and could help distinguish between transcriptional upregulation and post-transcriptional modifications. Overall, our findings indicate that iNOS plays a significant role in glucose-induced inflammation in DR, but additional mechanistic studies are necessary to disentangle the osmotic and metabolic contributions to its regulation.

*IL-6* expression was examined by qRT-PCR and was increased in the Glc25 group compared with that in the osmotic and native controls. This indicates the activation of the immune response to glucose stress. The increased *IL-6* expression in Glc25 compared to that in controls was consistent with previous scientific findings on the inflammatory processes in diabetic retinopathy. At the same time, in addition to its role in inflammatory processes, IL-6 also plays a crucial role in neoangiogenesis. Gopinathan et al. showed that IL-6 had an effect comparable to that of VEGF on angiogenesis induction in in vitro experiments [29]. Therefore, IL-6 should be given special attention in future studies, as its functions make it a link between inflammatory and microangiopathic pathophysiology.

IF staining revealed increased GFAP levels in Müller cells in the Glc25 group compared to those in the corresponding osmotic and native controls. These results indicated that glucose treatment resulted in the activation of Müller cells. In contrast, qRT-PCR showed reduced *GFAP* expression in the Glc25 group compared to Man25 and the control group. In 2017, Wang et al. showed that Müller cells survive high-glucose concentrations, re-enter the cell cycle upon treatment with glucose, exhibit irregular morphology, and migrate to other layers [20]. Cells with migrated nuclei showed reduced glutamate synthase in histological studies, indicating an immature differentiation state [20]. These Müller cells can become proliferative multipotent neuroprogenitors, which are then transformed into retinal neurons to replace the retinal cell types that remain in the DR [30,31,32].

In summary, there was an overexpression of GFAP in retinal explants as evidenced by immunofluorescence.

During qRT-PCR, it is possible that the increased expression of cytokines from Müller cells had already decreased and shifted to microglia, resulting in reduced expression of GFAP. Alternatively, Müller cells may have transformed into proliferative, multipotent neuroprogenitor cells due to glucose treatment, losing their typical glial cell characteristics, such as GFAP expression. However, it should be noted that a publication by Kuehn et al. in 2017 showed that GFAP expression tends to remain stable in the porcine ex vivo retina model under induced cell stress [33]. The present results do not allow for conclusive interpretation, which is why the experiments should be repeated at different time points to understand the time course. Because of the key role of glial cells, especially Müller cells, in DR, special attention should be paid to these cells in future studies.

**Figure 5:**
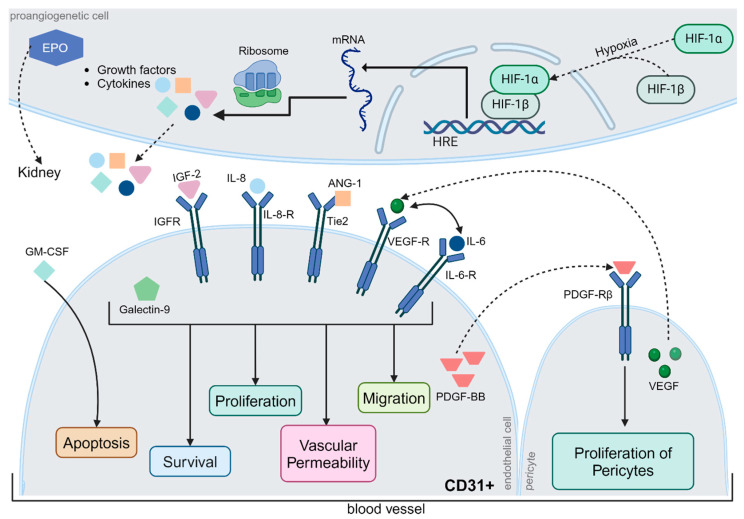
Regulation of Angiogenesis. Typically, neoangiogenesis is triggered by hypoxia, whereby hypoxia-inducible factor (HIF)-1α and HIF-1β form a complex that induces the expression of hypoxia-responsive transcription factors. The relevant gene segments were transcribed, translated, and released from the cells. Some proteins, such as erythropoietin (EPO), are transported via the bloodstream to the kidneys. Others act locally on surrounding blood vessels. Insulin-like growth factor-2, interleukin-6 and -8, angiopoietin-1, VEGF, and galectin-9 jointly affect cell survival, endothelial cell proliferation, vascular permeability, and migration via different metabolic pathways. In contrast, granulocyte-macrophage colony-stimulating factor (GM-CSF) exerts a pro-apoptotic effect on CD31+ endothelial cells. Platelet-derived growth factor (PDGF-BB) has a proliferative effect on pericytes, which are extremely important for physiological neoangiogenesis. However, the precise regulation of angiogenesis is still poorly understood, although the interactions between different metabolic pathways have been studied in detail. Abbreviations: ANG-1, angiopoietin-1; CD31, cluster of differentiation 31; EPO, erythropoietin; GM-CSF, granulocyte-macrophage colony-stimulating factor; HIF, hypoxia-inducible factor; HRE, hypoxia response elements; IGF, Insulin-like growth factor; IL, interleukin; mRNA, messenger ribonucleic acid; PDGF-BB, platelet-derived growth factor; R, receptor; Tie2, TEK receptor tyrosine kinase; VEGF, vascular endothelial growth factor. The figure was created by using https://www.biorender.com/ accessed 12 December 2024.

In the qRT-PCR evaluation, increased expression of *CD31* was observed in Glc25 compared to that in the native and osmotic control groups. Abu El-Asrar et al. were able to histologically demonstrate increased CD31-positive vessels, so-called neo-vessels, in active proliferative DR, which suggests an overexpression of CD31 in neovascularization in DR [34]. It is known that specialized endothelial cells can invade and migrate into tissue and thus represent an important part of angiogenesis [34]. Furthermore, the literature describes that during the course of DR, damaged endothelial cells can take on the characteristics of myofibroblasts and lose endothelial cell markers, such as VE-cadherin and CD31 [35,36,37]. Lin et al. first referred to this phenomenon as an endothelial-mesenchymal transition and characterized the transition to a differentiated cell type that confers invasive and migratory capabilities to cells that influence pathological processes [36]. In the late stage of DR, endothelial cell loss can be observed, although in 2008, Busik et al. showed that endothelial cells do not respond directly to elevated glucose concentrations but indirectly to cytokines, such as IL-1β or TNF-α, produced by neighboring cells (Figure 5) [2,9,38].

In summary, IL-6, TNF-α, iNOS, GFAP and CD31 are upregulated during treatment with glucose. IL-6, TNF-α and iNOS are closely linked to the NF-κB pathway that additionally induces other proinflammatory mediators, which in turn further increase ROS production (Figure 1). These combined mechanisms lead to DNA damage and apoptosis [6]. The elevated detection of GFAP is an indicator of increased activity or an increased presence of Müller cells. Along with microglia and astrocytes, Müller cells are the main producers of pro-inflammatory cytokines in the early phase of inflammation [39,40]. CD31 is an endothelial cell marker, and increased detection may indicate neoangiogenesis [34].

### 3.2. Limitations

These results, especially in the context of a novel porcine diabetic ex vivo retinal model, are promising. However, this study has some limitations that must be considered. One of the main limitations of an ex vivo model is the tight time frame, up to a maximum of eight days, whereas in vivo models can be bred for several weeks or even months. Because of the limited time, a high concentration of glucose was chosen, which recreates poorly adjusted diabetes mellitus with peaks in blood glucose concentration. Therefore, the experiments could be followed by a series of measurements over different incubation periods and how the specific inflammatory markers change. Further questions would be when the first measurable change in the respective inflammatory markers occurs or at what point the duration of the model is exhausted so that no more differences in induced cell death can be detected in the native control and the glucose group.

Moreover, DR in vivo is not due to direct contact with high-glucose levels on the retina, but through an elevated blood glucose level. Owing to the lack of blood circulation in an ex vivo model, the exact process cannot be recreated. Nevertheless, in 2014, Oshitari et al. described a small direct effect of increased permeability owing to a blood–retinal barrier breakdown [18]. However, the direct application of high-glucose concentrations to the tissue in the present model is not pathophysiologically correct. Studies by the same research group have shown that the results of ex vivo models can be transferred well to those of in vivo models [18]. Nonetheless, the breakdown of the blood–retinal barrier, in conjunction with osmotic effects, plays a crucial role in the pathogenesis of DR. These phenomena are not adequately represented in this ex vivo model.

In this study, the osmotic control groups should exclude sole osmotic effects. However, in vivo, these effects play a significant role in DR [41]. The interpretation of results from osmotic control can be challenging, and the extent to which osmotic effects influence inflammation remains uncertain. To address this knowledge gap, further experiments are necessary. These experiments should include glucose and osmotic control concentrations adjusted for different time points during the course of the disease. The results of these experiments could provide more differentiated statements about the interaction of osmotic effects and the effects of glucose treatment. Owing to the lack of embedding in tissues, apoptotic cells and other cellular components are more difficult to degrade and accumulate [42]. This can lead to a chain reaction in which damaged apoptotic cells or cell remnants further intensify the immune reaction, leading to fulminant inflammation that is not primarily triggered by treatment with stressors. Simultaneously, neighboring cells also influence each other in vivo, specifically through paracrine secretion and in an untargeted manner, such as through necrotic cell components. Therefore, its influence on neighboring cells should be considered in an ex vivo model, as it reflects the pathophysiology of the embedded tissue.

While the examination of contiguous tissue offers advantages with regard to the combined effects of the different cell types, examinations, such as quantitative real-time polymerase chain reaction (qRT-PCR) or Western blot, cannot differentiate between the individual cell types. A distinction between the cell types can only be made in a histological examination. To be able to make detailed statements about which cell type produces different inflammatory markers at which point in time, single cell culture models would be necessary. However, it should be noted that these models have other limitations than the ex vivo model that has been presented here.

During glucose treatment, an inflammatory response was induced in ex vivo retinal explants with all tested inflammatory markers. In six out of seven markers for DR, there was a significant difference between the osmotic control and glucose treatment. In the remaining experiments, there was still a difference between the native control and Glc25 groups. These findings not only fit well into the existing scientific context, but also imply changes due to treatment with glucose and, therefore, an immunological response to DR.

Despite the fact that an inflammatory immune response could be triggered by glucose treatment in the presented ex vivo model, there is potential for changes and improvements. As already indicated, further concentrations and time points could be analyzed in order to keep the osmotic effects as low as possible and, at the same time, identify the greatest possible presence of inflammatory markers. The course of concentration of the pro-inflammatory cytokines could also be investigated. A further possibility to improve the model would be the inclusion of RPE cells. Additionally, a chromophore shortage due to the missing RPE might influence photoreceptor degeneration [43]. One option to counteract this effect would be retinol supplementation. However, since in most pig organ culture models, retinoid is not supplemented [44], for comparison reasons, retinoid was not supplemented. The explants were treated in a dark chamber in the incubator. Alternatively, a circadian light rhythm could be included, and the effects of light on the retinal explants could be analyzed.

In summary, we were able to present a good model, although there are many different ways to further develop and improve the model. The ultimate goal would be to prospectively test therapeutic options on the model by adding potentially protective or therapeutic substances to a glucose group and then investigating whether there is a reduction in cell damage or reduction in inflammation.

## 4. Materials and Methods

### 4.1. Culture Preparation

The preparation and cultivation methods have been described in detail by Hurst et al. 2017 [45]. Porcine eyes were collected from a local abattoir, transported on ice, and immediately processed. The eyes were disinfected and opened under sterile conditions, and retinal explants without the RPE layer were obtained using a biopsy punch (Ø = 8 mm; Cat. No. 48801; Pfm Medical AG, Cologne, Germany) in the visual streak [23,46,47,48]. The explants were transferred onto 12-Well-Inserts (12-Well Insert PET, cellQART, Northeim, Germany, # 9310402) filled with culture media (see Table 1) and incubated for approximately 3 h (37 °C, 5.0% CO_2_; Cell culture cabinet/CO_2_-incubator, Heraeus, Hanau, Germany). Afterward, the explants were treated in five different experimental groups (Control (Co), D-Mannitol 25 mM (Man25), D-Mannitol 50 mM (Man50), D-Glucose 25 mM (Glc25), D-Glucose 50 mM (Glc50)) with the different agents added to the culture media and D-Mannitol serving as osmotic control and D-Glucose as diabetic groups. After treatment, the samples were returned to the incubator, where they were cultivated without light. The medium was changed after two days, and the retinal explants were harvested for further analysis on day four. The effects of the treatments were investigated using TUNEL analysis, IF, and qRT-PCR.

### 4.2. TUNEL

For histological evaluation, the explants were frozen in Tissue-Tek Medium (Cat. No. 6502; Fisher Scientific GmbH, Schwerte, Germany) in liquid nitrogen and placed in a freezer for a minimum of one night. To obtain cross sections, the explants were cut into 12 µm thick slices using a microtome (Leica, Wetzlar, Germany, CM1950).

The TUNEL Kit (In Situ Cell Death Detection Kit, TMR red, Cat. No. 12156792910; Roche Diagnostics GmbH, Mannheim, Germany) was used according to the manufacturer’s given instructions.

### 4.3. Immunofluorescence

The sections were fixed with ice-cold acetone for ten minutes and rinsed with Phosphate buffer solution before staining. The primary antibodies were diluted in a 5% bovine serum solution and incubated overnight at 5 °C in a refrigerator (see Table 2).

The next day, samples were incubated with secondary antibodies labeled with Alexa Fluor^®^ 488 or Alexa Fluor^®^ 555. A 0.01 μg/mL (Table 2). DAPI solution (Cat. No. 18860.01; SERVA Electrophoresis GmbH, Heidelberg, Germany) was applied for five minutes to stain nuclei. For immunohistochemical staining, corresponding negative controls were used.

### 4.4. Histological Evaluation

Three slices were examined for each explant and one photograph was taken from each section using a fluorescence microscope (ZEISS Axio Imager 2, Oberkochen, Germany). For the TUNEL images, ImageJ (1.53 k, Wayne Rasband and contributors, National Instiutes of Health, USA) software was used to count the number of DAPI+ nuclei per layer and TUNEL+ staining per layer. IF was investigated by measuring the intensity of the immunofluorescence signal after subtraction of the background intensity and normalization to the area of the inner nuclear layer, which was measured by converting the images into grayscale, background subtraction, and threshold determination. The area was quantified using the ImageJ macro.

### 4.5. qRT-PCR

mRNA was isolated from frozen retinal explants and reverse-transcribed using a MultiMACS™ mRNA and cDNA Synthesis Kit on a Multi MACS M96 Separator (Cat. No. 130-094-410; Miltenyi Biotec, Bergisch Gladbach, Germany) according to the manufacturer’s instructions. Retinal tissues were stored directly in 900 µL lysis buffer provided by the Miltenyi µMACS mRNA Isolation Kit until further processing. The first step involved lysing the tissue, followed by the addition of magnetic beads with poly(dT) tails to selectively bind mRNA. These beads were then captured using a magnetic column. Within the same magnetic field, reverse transcription was performed directly on the beads to synthesize cDNA. cDNA was eluted with 50 µL total volume. The concentration of the cDNA was determined using an Infinite^®^ 200 PRO device (Tecan Trading AG, Männedorf, Switzerland). The values were determined in duplicate with 2 µL cDNA per measuring point using the 260/280 nm ratio. Beforehand, the calibration was carried out using the elution buffer. The cDNA was then diluted using demineralized water to a concentration of 1 ng/µL for further use in qRT-PCR. The final concentration of each primer was 100 nM. For the reaction mix, 1 ng/μL cDNA was used in a reaction volume of 20 μL. qRT-PCR was performed using Universal SYBER Green Super mix on a thermal cycler (Cat. No. 1725271; BioRad Laboratories GmbH, Munich, Germany). The run consisted of 40 cycles, and each sample was analyzed in duplicate. The relative expression of target genes in the Man25, Glc25, and control groups was calculated using the 2-ΔΔCt method [49] and expressed as a fold-change in gene expression. The cDNA expression values of the investigated genes (Table 3) were normalized to the cDNA values of the housekeeping genes encoding β-actin and receptor-like protein 4 (RLP4), which served as the controls.

### 4.6. Statistical Analysis

For statistical analysis, one-way ANOVA with subsequent post hoc analysis using Dunnett’s or Bonferroni’s test (significance level α = 0.05) was performed using the statistical software GraphPad PRISM Version 5.01 (Boston, MA, USA). This allowed differences to be determined in comparison to the control (Dunnett’s test) as well as between the individual groups (Bonferroni). Three significance levels were used to describe different magnitudes of differences between the treatments: *p* < 0.05 (probability of error less than 5%), *p* < 0.01 (probability of error less than 1%), and *p* < 0.001 (probability of error less than 1‰).

## 5. Conclusions

As observed in murine models, treatment with glucose in this novel porcine ex vivo retina model increased apoptosis, particularly in the INL and ONL. Treatment was with glucose-induced retinal inflammation, which was demonstrated by the increased expression of inflammatory markers, such as iNOS, TNF-α, and IL-6. Cell markers showed an increased presence of endothelial cells. The findings in glial cells indicated an increase in primary cells and, over time, a decrease in the expression of glial cell markers. Therefore, this novel ex vivo retinal model is suitable for further investigation of DR and therapeutics, as well as an alternative to in vivo animal models.

## Figures and Tables

**Table 1:** Composition of the retinal culture media.

Reagent	Quantity
Neurobasal^TM^-A Medium	50 mL
N2-Supplement (1X)	500 µL
B-27™ Supplement minus Insulin	1 mL
Ciliary Neurotrophic Factor (CNTF)	0.5 µL
Brain-derived neurotrophic factor (BDNF)	0.5 µL
Penicillin/Streptomycin	500 µL
Gentamicin	50 µL

Initially, a stock of 1 M D-Glucose-Dilution and 1 M D-Mannitol-Dilution was prepared in demineralized water. A sterilization filter (Millex-HA Filter, 0,45 µm, Millipore) was used.

**Table 2:** Primary and secondary antibodies used for immunofluorescence.

Primary Antibodies			
Name	Species	Manufacturer	Dilution
GFAP	Mouse	BD Pharmigen, Heidelberg, Germany, # 556330	1:100
TNF-α	Goat	Santa Cruz, Eugene, OR, USA, # sc-1348	1:100
iNOS	Rabbit	ThermoFischer, Waltham, MA, USA, # PA1-036	1:50
**Secondary Antibodies**			
**Name**	**Species**	**Manufacturer**	**Dilution**
Alexafluor 488	Goat, anti-mouse	Invitrogen, Waltham, MA, USA, # A11001	1:1000
Alfexafluor 488	Donkey, anti-rabbit	Invitrogen, Waltham, MA, USA, # A21206	1:100
Alexafluor 555	Donkey, anti-goat	Abcam, Camebridge, Great Britain, # ab150130	1:50

Abbreviations: GFAP, glial fibrillary acidic protein; TNF-α: Tumor necrosis factor α; iNOS, inducible nitric oxide synthase.

**Table 3:** Gene primer for qRT-PCR.

Gene	Forward 5′–3′	Reverse 3′–5′
*b-Actin*	GGAGTCTCTCCGATCTGTGC	ATCGGGGAAGAAAGGACAGT
*RLP4*	CAAGAGTAACTACAACCTTC	GAACTCTACGATGAATCTTC
*IL-6*	CACCAGGAACGAAAGAGAGC	GTTTTGTCCGGAGAGGTGAA
*iNOS*	TGTTCAGCTGTGCCTTCAAC	CAGAACTGGGGGTACATGCT
*TNF-* *α*	CCACCAACGTTTTCCTCACT	CCAAAATAGACCTGCCCAGA
*GFAP*	GGAGAAGCCTTTGCTACACG	GGAGAAGCCTTTGCTACACG
*CD31*	CATTTCCAAAGTCAGCAGCA	CATCATCATGCCTCCCTTCT

Abbreviations: RPL4: Receptor-like protein 4, IL-6: Interleukin-6, iNOS: inducible nitric oxide synthetase, TNF: Tumor necrosis factor, GFAP: glial fibrillary protein, CD31: Cluster of Differentiation 31.

## Data Availability

Complete dataset available on request from the authors.

## Abbreviations

The following abbreviations are used in this manuscript:

CD31	Cluster of Differentiation 31
Co	Control
DAPI	4′-6-diaminodino-2-phenylindol-staining
DM	Diabetes mellitus
DR	Diabetic retinopathy
ELAM-1	endothelial-leukocyte adhesion molecule 1
EPO	Erythropoietin
GCL	Ganglion cell layer
GFAP	Glial fibrillary protein
Glc25	25 mM D-Glukose group
Glc50	50 mM D-Glukose group
GM-CSF	granulocyte-macrophage colony-stimulating factor
gp130	Glycoprotein 130
HIF	Hypoxia-inducible factor
ICAM-1	Intercellular adhesion molecule 1
IF	Immunofluorescence
IFN-γ	Interferon-γ
IKKβ	inhibitor of nuclear factor kappa-B kinase subunit beta
IL	Interleukin
INL	Inner nuclear layer
iNOS	Inducible nitric oxide synthetase
IPL	Inner plexiform layer
JAK	Januskinase
Man25	25 mM D-Mannitol group
Man50	50 mM D-Mannitol group
mRNA	Messenger RNA
NF-κB	nuclear factor ‘kappa-light-chain-enhancer’ of activated B-cells
ONL	Outer nuclear layer
OPL	Outer plexiform layer
PDGF-BB	Platelet-derived growth factor
qRT-PCR	Quantitative real-time polymerase chain reaction
RLP4	Receptor-like protein 4
STAT	Signal transducer and activator of transcription
TNF-α	Tumor necrosis factor α
TUNEL	Terminal deoxynucleotidyl transferase dUTP nick end labelling
VCAM-1	vascular cell adhesion protein 1
VEGF	Vascular endothelial growth factor
